# Single compounds elicit complex behavioural responses in wild, free-ranging rats

**DOI:** 10.1038/s41598-018-30953-1

**Published:** 2018-08-22

**Authors:** Michael D. Jackson, Robert A. Keyzers, Wayne L. Linklater

**Affiliations:** 1 Centre for Biodiversity & Restoration Ecology, Victoria University of Wellington, Wellington, New Zealand; 2 School of Biological Sciences and Centre for Biodiversity and Restoration Ecology, Victoria University of Wellington, Wellington, New Zealand; 3 School of Chemical and Physical Sciences and Centre for Biodiversity and Restoration Ecology, Victoria University of Wellington, Wellington, New Zealand

## Abstract

There is mounting evidence that single compounds can act as signals and cues for mammals and that when presented at their optimal concentration they can elicit behavioural responses that replicate those recorded for complex mixtures like gland secretions and foods. We designed a rapid bioassay to present nine compounds that we had previously identified in foods, each at seven different concentrations (63 treatments), to wild, free-ranging rats and scored each treatment for attraction and three behavioural responses. Nine treatments (taken from five compounds) statistically outperformed the current standard rat attractant, peanut butter. Attraction to treatments was highest at the two lowest concentrations (0.1 and 0.01 μg g^−1^) and a statistically significant relationship of increasing attraction with decreasing treatment concentration was identified. Our study identified five compounds not previously associated with behavioural responses by rats that elicit equivalent or more intense behavioural responses than those obtained with peanut butter. Moreover, attraction to treatments was driven by a concentration-dependent relationship not previously reported. This is the first study to identify isopentanol, 1-hexanol, acetoin, isobutyl acetate and 2-methylbutyl acetate as possible semiochemicals/cues for rats. More broadly, our findings provide important guidance to researchers in the ongoing search for mammalian semiochemicals and cues.

## Introduction

Olfaction is the oldest and frequently the most important sense for animals^1^. Through sensing, processing, translating and interpreting thousands of different chemical signals in their environment, animals can regulate social and physiological behaviours, like mate-finding and reproduction, and locating food^2–4^. For most mammals, and especially rodents, olfaction is their primary sense, but despite its importance it remains one of the least understood senses^4,5^. For example, the mechanisms by which odours induce innate behavioural responses in mammals remains largely unknown^6^ and to-date only a small number of mammalian semiochemicals (a chemical substance that transmits a signal) have been formally characterised^7^.

Odouriforous products, be they urine, faeces, gland secretions, body odours, and foods, are complex natural mixtures. Australian brushtail possum cloacal secretions, for instance, contain >100 compounds across a range of different chemical classes^8^. This complexity has led to suggestions that mammalian olfaction may be primed to identify and interpret complex mixtures^7,9–11^. For instance, although olfactory receptors have compound specificity, intra-species communication is commonly suggested to be driven by combinations or “bouquets” of odours that work interactively as a whole^12,13^. However, of the formally characterised semiochemicals or cues with known signalling roles in mammals, many are single compounds^14^.

Single compound semiochemicals and cues have been reported to elicit an array of behavioural responses in mammals like biting and chewing responses in canids and felids^15^, attraction, aversion and inter-male agression in mice^6,16–18^ and aversion, anxiety, mother-young development primers and female attraction in rats^19–21^. In some cases the behavioural responses to the single compound was as strong as for the complex mixture in which it was identified. Further, some olfactory sensory neurons can be triggered by single compounds, thus it is possible they elicit innate behavioural responses^22^. However, studies investigating olfactory-mediated behavioural responses to semiochemicals and cues are almost exclusively performed using laboratory-bred animals (e.g., Wistar rats or house mice) or captive animals held in pens, not free-ranging wild animals. This has important implications as the results obtained from wild, free-ranging animals are likely to provide more realistc “real world” outcomes as they discover, test and confirm animal responses across an enormous range of natural conditions. Thus, compounds can be presented to wild animals in a complex ‘odourscape’ of myriad competing olfactory signals and cues with different meaning or function. This means that any compound(s) that stand out from the ‘chemical cacophony’ and are detected by the target species may be important communicatory signals^23^. Further, replication at the multi-population (site) scale is possible compared with finite captive (pen or laboratory) animals, thus allowing for broader, species-level inferences^24,25^.

The search for semiochemicals and cues, whether single compounds or blends, is made difficult by a range of species- and compound-specific and environmental factors that have the capacity to significantly impact their identification. Firstly, an animal’s behavioural response to a compound is commonly concentration-dependent^26^. For example, rats are attracted to carbon disulphide, dimethyl disulphide and dimethyl sulphide at 50 μg g^−1^ but repelled at 100 μg g^−1 ^^27^. Secondly, an animal’s detection threshold to compounds is also compound-dependent^28,29^. Rats have a detection threshold of 0.0001 ng g^−1^ for 2,4,5-trimethylthiazoline, a predator odour that elicits fear in rats^30^, but a threshold of just 1 μg g^−1^ for some aliphatic esters^31^; a difference of seven orders of magnitude. This heightened olfactory sensitivity to a compound(s) may provide important information about the behavioural relevance of that compound to the study animal^32,33^. Thirdly, a compound’s molecular weight, vapour pressure and a suite of environmental factors directly impact signal propagation. Advection and turbulence can lead to patchy odour-active spaces (the space in which the compound is at, or above, the animals detection threshold), while wind can dramatically decrease the concentration of the compound at or near its source^14,28,34,35^. Lastly, in-field studies are often subject to a range of logistical constraints. For example, monitoring devices like camera-traps are expensive to buy while their bulk, weight and installation time means relatively low numbers of treatments can be assayed at the same time. Further, scoring videos for behaviours is time consuming, therefore costly. Thus, studies aiming to identify signals or cues using wild, free-ranging animals must cope with extraordinary chemical, environmental and animal variance, and ensure that large numbers of treatments are subjected to within and between site replication in a balanced assay design.

We devised a rapid, highly replicable, field-based assay using tracking tunnels that are used internationally to monitor rats^36–40^ and have previously been used assess the attractiveness of compounds on wild rats^41^. This allowed us to overcome the enormous environmental, species- and compound-specific issues associated with presenting compounds to wild, free-ranging animals and ensured we could assay the large number of treatments we intended to present in a robust, balanced design. We used this bioassay to present nine compounds that we identified as having the potential to act as signals/cues for rats, each at seven different concentrations (63 treatments). The nine compounds were identified following rapid, in-field bioassays that presented a range of food products to rats, the chemical profiling of the foods using headspace solid-phase microextraction gas chromatography-mass spectrometry (HS-SPME GC-MS) and the statistical interrogation of the GC-MS dataset using partial least squares regression^42,43^.

Our study objectives were to: (1) quantify the attractiveness of each compound on wild, free-ranging rats. We hypothesised that several of the nine compounds would be attractive to rats and that attraction to those compounds may be as strong as for a complex mixture such as peanut butter, a product that is widely used to lure rats; (2) quantify a range of behavioural responses to the compounds that may provide information about the compounds’ importance to rats. We hypothesised that attractive compounds were also likely to elicit behaviours such as biting and urine marking and; (3) identify any concentration-dependent relationships between the treatments and each behavioural response. Given the olfactory sensitivity of rats to behaviourally important compounds and that concentration-dependent responses to some compounds has been reported in rats and other species, we hypothesised that a relationship between one or more of the behavioural responses and the treatment concentration was likely.

## Results

### Trial eliminations – attraction

Forty-two of the 63 treatments presented during Phase One (*n* = 5 per treatment) received a confirmed rat visit. Treatment I7 was the most attractive treatment with an attraction rate of 0.80, while A6, B6, I6 and C7 were the next most attractive treatments, each with an attraction rate of 0.60. These top five most attractive treatments were all presented at the two lowest concentrations: 0.1 and 0.01 μg g^−1^. The mean attraction rates for the control and peanut butter standard were 0.20 and 0.26, respectively. Forty-eight of the 63 treatments presented in Phase One were statistically weaker than the most attractive treatment I7 (P ≤ 0.01) and were therefore excluded from Phase Two trials, except for D1, D6, E3, E7, F3, G2, G6, and H7 and I1 that were carried forward to Phase Two trials in-line with our methodology. In total, twenty-seven treatments were carried forward to Phase Two, with a minimum of two representatives from all nine compounds and all seven concentrations.

In Phase Two, the 27 treatments carried forward from Phase One were presented (*n* = 5 per treatment for Phase Two and thus *n* = 10 in total for the 27 compound-based treatments presented in Phase One and Two). Treatment F7 was the most attractive treatment after Phase Two, with an attraction rate of 0.50 (*n* = 10). Treatment I7 was the second-most attractive, with an attraction rate of 0.40 (*n* = 10). The mean attraction rates for the control and peanut butter standard were 0.13 (*n* = 70) and 0.16 (*n* = 70), respectively.

**Figure 1:**
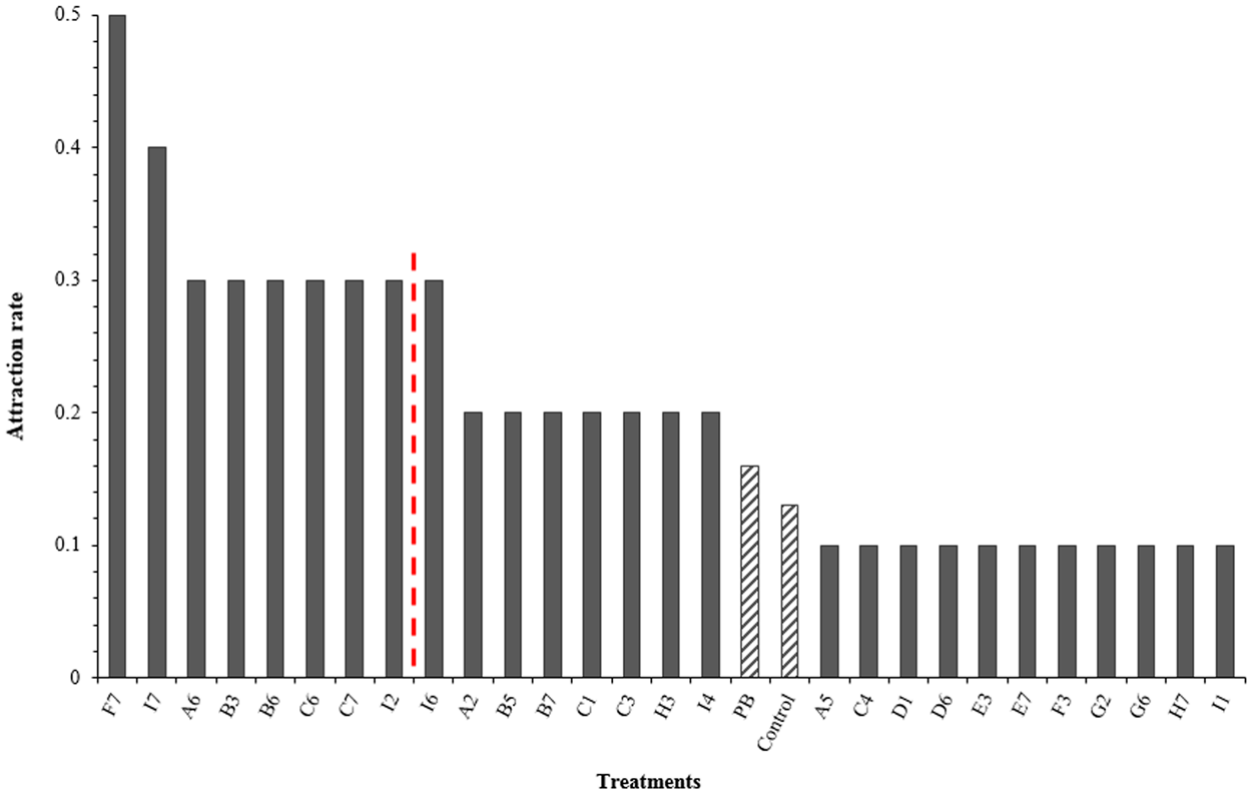
Attraction rate for treatments presented in both Phase One and Two (*n* = 10). The mean attraction rate for the control and peanut butter standard (PB) were 0.13 and 0.16, respectively, and are shown hatched to provide differentiation from treatments. Treatments to the left of the dotted line statistically outperformed the peanut butter standard and the control (*P* < 0.01).

Sixteen treatments were more attractive than peanut butter after the 10 trials, nine of which were significantly more attractive (*P* < 0.01; A6, B3, B6, C6, C7, F7, I7, I2, I6 and I7: Fig. 1). Of the nine significantly more attractive treatments, seven were presented at the two lowest concentrations of 0.1 and 0.01 μg g^−1^. The nine significantly more attractive treatments were derived from five compounds: isopentanol (A); 1-hexanol (B); acetoin (C); isobutyl acetate (F) and 2-methylbutyl acetate (I). A trial-by-trial breakdown of the results is provided in Supplementary information S1.

### Biting, marking and investigation

**Figure 2:**
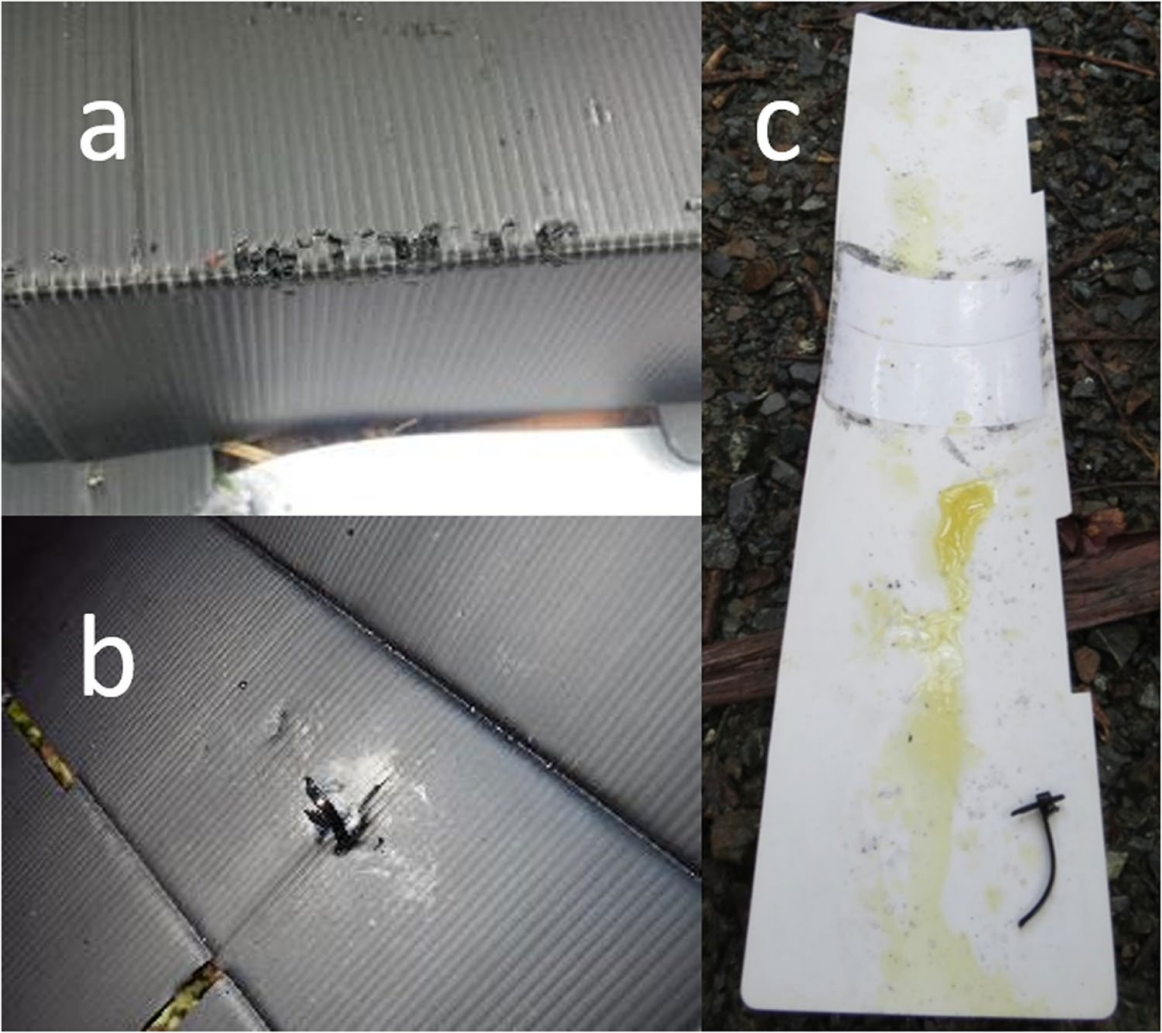
Examples of biting and marking events with treatments (**a**) extensive biting of the tracking tunnel roof, (**b**) biting of the cable tie that held the microtube. The microtube was missing from this tunnel upon inspection and, (**c**) extensive urination on the tracking card inside the tracking tunnel. The cable tie was also bitten and the microtube containing the treatment was missing. Photos by MDJ.

Twelve of the 27 treatments presented in Phase Two received a biting event. Seven of the nine treatments that were significantly more attractive than peanut butter (A6, B6, C6, C7, F7, I6 and I7) received a biting event and these were all presented at the two lowest concentrations (0.1 and 0.01 μg g^−1^). The two statistically significant treatments that did not receive a biting event were B3 and I2 (presented at 100 and 1000 μg g^−1^, respectively). Peanut butter had the highest biting rate (0.73), but only statistically outperformed three of the nine statistically significant treatments: I2 and B3 that received no biting (P = 0.006 for both treatments) and F7 that received only one biting event (P = 0.02). The control received no biting events. A range of different biting responses were recorded (Fig. 2).

Marking on or in tracking tunnels was recorded for 18 of the 27 treatments (Fig. 2). Treatment I2 (presented at 1000 μg g^−1^) was the only one of the nine significantly attractive treatments not to receive a marking event. Six of the nine significantly attractive treatments scored a higher marking rate than peanut butter (PB marking rate 0.36), with four presented at the two lowest concentrations receiving significantly more marking events than peanut butter (A6, C6, C7, P = 0.04; I6, P < 0.001). The control received no marking events.

Treatment A2 had the highest mean investigation score (195, SE ± 11) while peanut butter had the second highest investigation score (164, SE ± 22.66). Conversely B5 (presented at 1 μg g^−1^) scored the lowest mean score (23, SE ± 4.5), with the control receiving a higher mean score (110, SE ± 22.96). No statistically significant difference between treatments, peanut butter and the control was identified (*H = *21.814 *df* = 17, P = 0.19). We only used assays with a minimum of two observations in our Kruskal Wallis analysis as the inclusion of a single observation would provide no variance. The data and R code for each test for each behavioural response are provided in Supplementary information S1.

### Concentration-dependent relationships

Attraction to treatments was highest at the two lowest concentrations, with nearly half of all recorded visits occurring with treatments presented at 0.1 and 0.01 μg g^−1^. The lowest recorded attraction rate (0.09) was for treatments presented at 1 μg g^−1^. A statistically significant relationship between treatment concentration and attraction was identified (*X*^2^_(GLMM)_ = 6.24, *df* = 1, P = 0.013).

Biting was only recorded at three concentrations: 0.01, 0.1 and 1000 μg g^−1^, therefore we did not perform a GLMM for biting.

Marking on or in tracking tunnels was recorded at each of the seven concentrations. Marking was highest at the two lowest concentrations, with 53% and 44% of all visits to treatments at 0.1 and 0.01 μg g^−1^, respectively, receiving some faecal and/or urine marking but no statistically significant relationship between treatment concentration and marking behaviour was identified (*X*^2^_(GLMM)_ = 1.90, *df* = 1, P = 0.17).

Investigation was highest for treatments presented at 0.1 μg g^−1^ and 100 μg g^−1^ (mean score for both was 134) while treatments presented at 1 μg g^−1^ scored the lowest for investigation (mean score 88). No statistically significant relationship between treatment concentration and investigation was identified (*X*^2^_(LMM)_ = 0.0, *df* = 1, P = 0.998). The data and R code for all models are provided in Supplementary information S1.

## Discussion

Our study demonstrates that wild, free-ranging rats can detect and respond to a suite of different single compounds, and that when the compounds are presented at optimal concentrations they can elicit levels of behavioural responses that outperform a complex mixture like peanut butter. Furthermore, some behavioural responses to our compounds appear to be concentration-dependent, with higher levels of attraction and marking rates recorded at lower concentrations and a statistically significant negative relationship between attraction and compound concentration identified.

Rats were able to respond to a range of different single compounds amid the cacophony of olfactory noise found within and between sites, and commonly responded to the compounds with complex behaviours like urine marking and/or biting. This strongly suggests that isopentanol, 1-hexanol, acetoin, isobutyl acetate and 2-methylbutyl acetate may be important communicatory signals or cues for rats. The high levels of behavioural responses to compounds presented at low concentrations further supports this assertion. As detailed in the introduction, an animal’s olfactory sensitivity to an odorant can provide important information about that odorants evolutionary and behavioural importance to the focal animal^44^. For instance, Norway rats *Rattus norvegicus* are several orders of magnitude more sensitive to 2,4,5-trimethylthiazoline, a predator odorant known to elicit fear in rats, than non-human primates for which it elicits no fear response^30^. Moreover, urine/faecal marking is a common response by rats to orientate and attract conspecifics to objects of importance^45,46^. We hypothesise, therefore, that isopentanol, 1-hexanol, acetoin, isobutyl acetate and 2-methylbutyl acetate may be behaviourally important semiochemicals or cues for rats given, (1) the level of attraction to the compounds across multiple independent populations, (2) the ability of rats to discern the compounds above each site’s olfactory noise and, (3) the low concentrations at which they elicited a behavioural response. To the best of our knowledge, none of the five compounds have been previously described as semiochemicals or cues for rats.

Some compounds elicited higher levels of urine marking than recorded for the peanut butter standard (e.g., compound A, C, and I for urine marking). This also raises the possibility that one or more of the five attractive compounds that we originally identified in foods^43^ may be pheromonal in nature as pheromones are commonly exaptation’s of compounds that originally had other uses, such as those derived from foods^47^. Intriguingly, three of the five top performing compounds (isopentanol, 1-hexanol and acetoin) have been reported in rat urine, although they have not been formally characterised as pheromones^48–50^.

Low attraction rates to higher concentrations may be due to the animal’s olfactory receptors interpreting the compounds differently or becoming fatigued through their saturation and ultimately leading to avoidance and/or repellence^27,51^. Thus, the perceived aroma of the compounds may have changed with increasing concentration. A human example is 4-methyl-4-sulfanylpentan-2-one that typically occurs in wines made with Sauvignon blanc grapes. Below 5 ng L^−1^ humans discern this compound as having an aroma of passionfruit, but it smells of cat urine at ca. 5 ng L^−1^ or above^52^. Moreover, some studies have shown that behavioural responses to odours are mediated by sensory neuron and glomeruli activation patterns that change dramatically depending on the concentration of the odour presented^53,54^. We hypothesise that these factors, allied with the olfactory sensitivity of rats to behaviourally important compounds, may explain the concentration-dependent relationship for attraction we report in this study.

Concentration-dependent attraction to compounds has been demonstrated for nematodes, fruit flies, humans and rabbits^53–55^. Those studies showed an initial increase in attraction to an olfactory stimulus with decreasing concentration until a peak response was achieved, after which attraction or the behavioural response began to decrease with decreasing concentration, creating a curve akin to a normally distributed response. This normally distributed outcome is also demonstrated by^56^ who measured the frequency of penile erections in Norway rats in response to a blend of compounds. An initial increase in the frequency of erections was recorded with decreasing concentration until peak response was obtained, after which the frequency declined. It is possible, therefore, we may not have identified peak attraction in rats, as our study did not present the compounds at concentrations lower than 0.01 μg g^−1^ (the concentration with the highest attraction rate). We suggest future studies should present the same compounds at concentrations lower than those used in this study as this will allow for the elucidation of the rat’s peak concentration response and may, therefore, help pin-point the optimal concentrations for attraction and the behavioural responses for each of these compounds.

Our study demonstrates that five single compounds can act as signals/cues for wild, free-ranging rats and that they can elicit behavioural responses such as attraction, urine/faecal marking and biting that outperform more complex mixtures like peanut butter. To the best of our knowledge, this is the first study to identify isopentanol, 1-hexanol, acetoin, isobutyl acetate and 2-methylbutyl acetate as signals/cues for rats and to demonstrate a statistically significant relationship between attraction to compounds and their concentration.

Given the importance of olfaction to mammals (Solomon *et al*., 2007) and the concentration-dependent relationship for attraction identified in this study, we suggest bioassays assessing behavioural responses of mammals to semiochemicals should initially consider presenting compounds at low concentrations, such as 0.1 μg g^−1^ or lower. That said, the physical properties of individual compounds, such as vapour pressure, and the animal’s detection threshold for that compound, may demand that a broad spectrum of concentrations are at least initially considered. Indeed, a broad-spectrum approach may ensure the subject animal’s peak response to a compound is found. Nonetheless, our findings suggest a bioassay that initially focuses on lower concentrations may prove a more fruitful approach to identifying behaviourally important semiochemicals, with higher concentrations trialled if attraction rates at low concentrations are poor. Further, given that behaviourally important compounds are likely to be discriminated at very low concentrations, consideration should be given to presenting compounds at concentrations lower than those presented during this study. Lastly, the identification of attraction to more than half of the compounds identified using partial least squares regression and detailed in our previous work^43^ provides demonstrable support for its use of our reductive statistical approach to identify single compounds that may act as cues or signals. This study provides evidence of the usefulness of our statistical response-guided approach to the identification of signals and cues for mammals, despite some authors suggesting it is not possible to simplify mammalian signal complexity using such a strategy^57^.

## Materials and Methods

### Treatment preparation and presentation

Treatments were prepared by serially diluting an initial stock solution of each compound in medium chain triglyceride oil (MCT) in 2 mL microtubes (Supplementary Table S2 for dilution procedure). We used MCT as prior GC-MS analysis identified it as having the lowest volatile profile when compared with two traditional carrier media, propylene glycol and glycerine. Microtubes (2 mL) were subjected to mixing using a Chiltern MT19 vortex mixer for 20 seconds. One gram of the final treatment was pipetted into a 1.7 mL microtube for in-field presentation^58–60^. Th microtube lids were sealed with Parafilm® and each treatment was placed in an individual, labelled zippered plastic bag and stored overnight at 4 °C. The 63 treatments (nine compounds at seven concentrations – see Trial Design for further detail) were prepared 24 hours prior to each trial.

**Figure 3:**
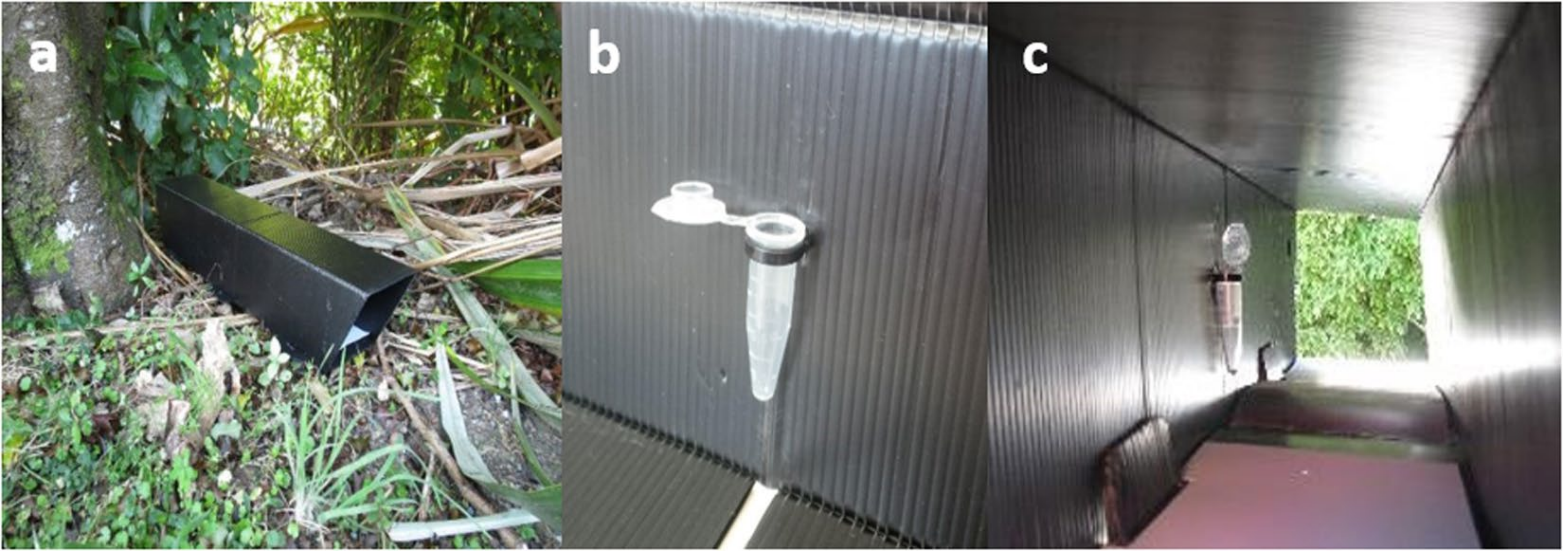
Tracking tunnel in-field set-up showing (**a**) *in situ* tracking tunnel, (**b**) microtube containing the treatment suspended on the inside wall of the tunnel using a cable tie and, (**c**) the internal structure of the tracking tunnel, with the treatment-containing microtube and inked tracking card visible. Photos by MDJ.

Treatments were presented to wild, free-ranging rats in pre-conditioned (washed with rainwater and left to air-dry outdoors for 2 weeks) tracking tunnels^39^. Treatments were secured to the inside wall of tunnels using a cable tie. Tracking cards, with non-drying ink applied to the centre of the card, were placed in each tunnel to quantify visits and allow for the identification of the species visiting the treatments (Fig. 3). Only four rodent species are present in New Zealand and of those, only three (*Rattus rattus*, *Rattus norvegicus* and *Mus musculus*) were present in the trial locations, thus allowing for accurate track discrimination.

Tracking tunnels were installed along a single spatially stratified transect that followed walking tracks for ease of accessibility, speed of tracking tunnel installation and safety during in-field work. Tracking tunnels were installed between 2 and 15 m (into the forest) from pathways and fixed to the ground using metal mat pins. Each tracking tunnel was constructed and deployed by the same operative wearing a new pair of single use gloves to avoid human scent transmission to tracking tunnels or microtubes, and in-field cross-contamination between tunnels from unpacking and handling treatments. Tracking tunnels were cleaned after each trial using tap water, rinsed with rainwater and left to air-dry outdoors. Tracking tunnels were assigned to individual, concentration-specific treatments for the duration of the trials. Tunnels that received rat interactions, such as extensive chewing and/or urine marking or those where the treatment was spilt due to rat interactions with the microtube were, however, not used in future trials and were replaced with new, pre-conditioned tunnels.

### Trial design

For Phase One trials, each of the nine compounds was presented at seven different concentrations, decreasing logarithmically from 10,000 to 0.01 μg g^−1^. This was because (1) the concentration range covered those previously trialled for rats and other vertebrate pest species^61–64^, (2) the relationship between odour intensity and concentration can be modelled by a log-linear relationship^65^, and (3) the approach allowed us to investigate the impact of concentration on the behavioural responses of rats to treatments.

The nine trial compounds were provided letter codes from A to I while the seven concentrations were coded from 1 to 7, thus providing a unique identifier for each of the 63 treatments. The nine compounds and their associated codes were: isopentanol (A); 1- hexanol (B); acetoin (C); isopentanoic acid (D); 2,3-dimethylpyrazine (E); isobutyl acetate (F); isopentyl acetate (G); tetramethylpyrazine (H) and 2-methylbutyl acetate (I). The seven concentrations and their associated codes were: 10,000 μg g^−1^ (1); 1000 μg g^−1^ (2); 100 μg g^−1^ (3); 10 μg g^−1^ (4); 1 μg g^−1^ (5); 0.1 μg g^−1^ (6) and 0.01 μg g^−1^ (7). Therefore, the unique identifier code A6, for example, would specify isopentanol at a concentration of 0.1 μg g^−1^. This provided 63 concentration-specific treatments. Individual treatments are hereafter referred to by their unique identifier code (Supplementary Table S3 for the full table of codes).

For Phase One, each trial was made up of seven strata installed along a single spatially stratified transect. Each stratum comprised treatments presented at the same concentration. Tracking tunnels containing treatments were spaced at 25 m, with strata separated by 200 m as these distances matched or exceeded the spacing’s used for previous in-field compound trials and current monitoring protocols for rats^39,41^. A control (MCT only) and standard (peanut butter) in 1.7 mL microtubes were assigned to each stratum. In total, 11 tracking tunnels (nine treatments, one control and one standard) were installed along each of seven strata (77 tunnels in total).

For Phase Two, the most attractive treatments identified at the end of Phase One trials were presented (see Data analysis for the statistical procedure used to identify the most attractive treatments). Each trial was made up of seven strata installed along a single spatially stratified transect. Each stratum comprised treatments presented at the same concentration. Treatments were spaced at 50 m, with strata separated by 200 m. This increase in spacing from 25 to 50 m was designed to allow us to incorporate greater spatial and population variance in our study. A control (MCT only) and standard (peanut butter) in 1.7 mL microtubes were assigned to each stratum.

The order of strata and that of the treatments within each stratum was randomised for each of the 10 trials. All treatments were left *in situ* for one rain-free night. Phase One trials (*n* = 5) were undertaken between 15^th^ July 2015 and 8^th^ September 2015 and Phase Two trials (*n* = 5) were undertaken between 15^th^ September 2015 and 11^th^ November 2015. Phase One and Two trials were run across the Greater Wellington region, North Island, New Zealand (Supplementary Figure S4 for map and site coordinates). This study was conducted with approval and in accordance with the Victoria University of Wellington Animal Ethics Committee – Approval number 22351.

### Response variables

Each treatment was scored for four behavioural responses: attraction, marking, biting and investigation. This is important as behaviour-specific responses to compounds may provide useful information regarding the behavioural importance of the compound to the focal species. Rats, for example, are known to deposit urine and faecal scent marks on or near foods or objects of interest that convey important information to conspecifics about the items presence and location^45,46^.

Attraction was scored using the presence/absence of rat tracks on inked tracking cards to provide a proportion of tracking cards presented for each treatment that received rat visits and hereafter termed the ‘attraction rate’. For example, an attraction rate of 0.50 means that half of the tracking tunnels installed containing a specific treatment received a confirmed visit. We also recorded (1) the presence (binary measure) of urination and/or faecal marking on or in the tracking tunnel and hereafter termed ‘marking’, (2) the presence of chew or bite marks on the microtube and/or tracking tunnel and hereafter termed ‘biting’ and, (3) the area tracked with footprints on each tracking card that received a visit and termed ‘investigation’. This was measured using a 10 × 47 cm Perspex sheet with a grid made up of 1 × 1 cm squares. The number of squares with rat tracks provided an investigation score that was designed to identify treatments that generated a strong response by an individual or that elicited visits from multiple individuals.

We used the attraction rate to direct the elimination process detailed in the Trial Design section. Treatments that had a statistically significantly lower attraction rate than the most attractive treatment after Phase One trials were eliminated and not carried forward to Phase Two trials. This allowed for a rapid removal of unattractive treatments. For completeness, however, and to avoid the possibility of false negative outcomes we applied the following additional criteria to the selection of treatments to be presented in Phase Two (1) if a statistically weaker treatment received either a marking and/or biting event that treatment was carried forward to Phase Two and, (2) if a compound had only one representative from all seven concentrations to be carried forward to Phase Two, an additional treatment of the same compound that had received a visit was included. The two rules ensured all nine compounds were carried forward to Phase Two trials with at least two representatives thus providing a more conservative criterion designed to mitigate the likelihood of false negative outcomes.

### Data analysis

We used binomial tests to compare the performance of each treatment to the most attractive treatment at the end of each in-field trial phase and to compare treatments to both the control and peanut butter standard. The attraction rate for each treatment was used to guide this process and to identify the most attractive treatment at the end of Phase Two. We also used binomial tests to compare marking and biting response rates for treatments with each other, the peanut butter standard and control. Differences in investigation between treatments, control and the standard were examined using a non-parametric Kruskal-Wallis ANOVA.

Concentration-dependent relationships between each response variable (attraction, biting, marking and investigation) and treatments combined based on concentration were examined using a Generalised Linear Mixed-Effects Model (GLMM) with a binomial distribution and logit link for attraction, marking and biting rate (as the response was binary). A Linear Mixed-Effects Model (LMM) was used for investigation (because the response was continuous). Both GLMM and LMM models were run with ‘Compound’ nested within ‘Site’ as a random effect structure. A Type 3 Wald test was used to obtain each model’s test statistic.

The model data were constructed by combining the data for all the treatments based on concentration. For example, the results for all nine treatments presented at 10,000 μg g^−1^ across the 10 trials were combined. We did this for all seven concentrations. This allowed us to identify whether attraction, biting, marking and investigation of rats to the compounds was potentially concentration dependent. Biting, marking and investigation models were run using data only obtained from tracking tunnels that received a confirmed visit. This allowed us to interrogate possible concentration-dependent relationships using data from verified visits. Statistical significance was assumed wherever *P* ≤ 0.05. All statistical analyses were run in R, version 3.1.3^66^, with package *lme4*^67^ used for mixed-effects models and *car*^68^ used for Type 3 Wald tests.

## Electronic supplementary material

Supplementary Information S2, S3 and S4

S1
