# Author Correction: Single compounds elicit complex behavioural responses in wild, free-ranging rats

**DOI:** 10.1038/s41598-018-32732-4

**Published:** 2018-10-05

**Authors:** Michael D. Jackson, Robert A. Keyzers, Wayne L. Linklater

**Affiliations:** 1 Centre for Biodiversity & Restoration Ecology, Victoria University of Wellington, Wellington, New Zealand; 2 School of Biological Sciences and Centre for Biodiversity and Restoration Ecology, Victoria University of Wellington, Wellington, New Zealand; 3 School of Chemical and Physical Sciences and Centre for Biodiversity and Restoration Ecology, Victoria University of Wellington, Wellington, New Zealand

Correction to: *Scientific Reports* 10.1038/s41598-018-30953-1, published online 22 August 2018

In Figure 1, the dotted line used to indicate the treatments that were statistically outperformed by the most attractive treatment (F7) is in the incorrect place. The current figure places the line between and I2 and I6 - this is incorrect. The line should be between I6 and A2. The correct Figure 1 appears below.

**Figure 1:**
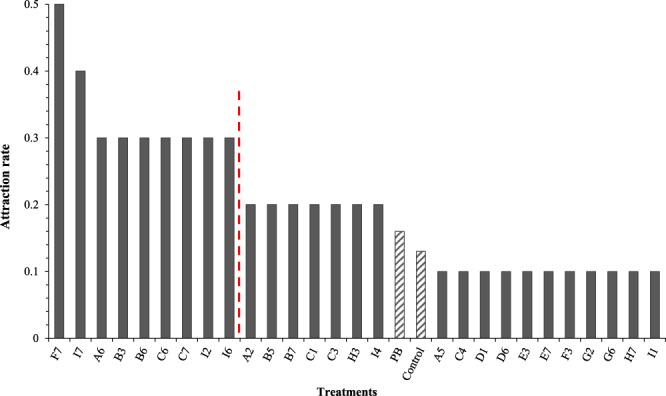
Attraction rate for treatments presented in both Phase One and Two (*n* = 10). The mean attraction rate for the control and peanut butter standard (PB) were 0.13 and 0.16, respectively, and are shown hatched to provide differentiation from treatments. Treatments to the left of the dotted line statistically outperformed the peanut butter standard and the control (*P* < 0.01).

