# Supplementary material for: Single compounds elicit complex behavioural responses in wild, free-ranging rats
Source: Sci Rep. 2018 Aug 22;8:12588. doi: 10.1038/s41598-018-30953-1 (PMC6105672; doi:10.1038/s41598-018-30953-1)
Supplement: Supplementary file 1 — Supplementary Information S2, S3 and S4 [file 41598_2018_30953_MOESM1_ESM.pdf]

Single compounds elicit complex behavioural responses in wild, free-ranging rats

Michael D. Jackson*, Robert A. Keyzers and Wayne L. Linklater

Centre for Biodiversity & Restoration Ecology, Victoria University, Wellington, New Zealand

*Correspondence to michael.jackson@vuw.ac.nz

Supplementary information

Supplementary table S2: Compound serial dilution method

Sample	ug g-1	Production
Standard solution	10000	0.015 grams compound in 1.485 grams MCT. Creates 1.5 grams of 10,000 ug g^{-1} standard solution
1	10000	Pipette 1 gram of the standard solution into microtube
2	1000	Pipette 0.15 gram of standard solution in 1.35 g MCT. Creates 1.5 grams of 1000 ug g^{-1} sample.
3	100	Pipette 0.15 gram of sample 2 in 1.35 g MCT. Creates 1.5 grams of 100 ug g^{-1} sample.
4	10	Pipette 0.15 gram of sample 3 in 1.35 g MCT. Creates 1.5 grams of 10 ug g^{-1} sample.
5	1	Pipette 0.15 gram of sample 4 in 1.35 g MCT. Creates 1.5 grams of 1 ug g^{-1} sample.
6	0.1	Pipette 0.15 gram of sample 5 in 1.35 g MCT. Creates 1.5 grams of 0.1 ug g^{-1} sample.
7	0.01	Pipette 0.15 gram of sample 6 in 1.35 g MCT. Creates 1.5 grams of 0.01 ug g^{-1} sample.

Supplementary table S3: Compounds unique identifier code provided each compound based on each of the seven concentrations trialled from 10,000 to 0.01 µg g^{-1}

**Compound**	**Compound Code**	**Concentration-specific code based on µg g^{-1}**						
		**10000**	**1000**	**100**	**10**	**1**	**0.1**	**0.01**
		1	2	3	4	5	6	7
Isopentyl alcohol	A	A1	A2	A3	A4	A5	A6	A7
1-Hexanol	B	B1	B2	B3	B4	B5	B6	B7
Acetoin	C	C1	C2	C3	C4	C5	C6	C7
Isopentanoic acid	D	D1	D2	D3	D4	D5	D6	D7
2,3-Dimethylpyrazine	E	E1	E2	E3	E4	E5	E6	E7
Isobutyl acetate	F	F1	F2	F3	F4	F5	F6	F7
Isopentyl acetate	G	G1	G2	G3	G4	G5	G6	G7
Tetramethylpyrazine	H	H1	H2	H3	H4	H5	H6	H7
2-Methylbutyl acetate	I	I1	I2	I3	I4	I5	I6	I7

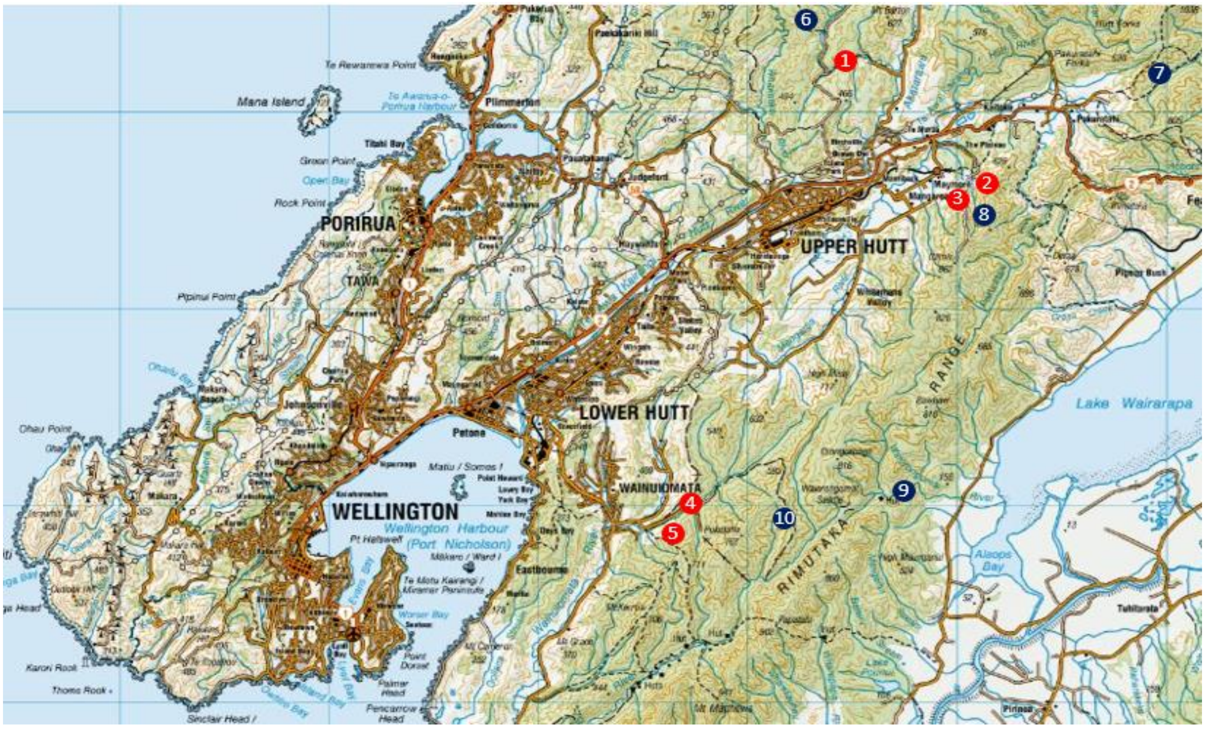

Supplementary figure S4: Phase One (red) and Phase Two (blue) trial sites across the Wellington Region, New Zealand. The site name and GPS coordinates are: Site 1 = Karapoti Gorge, Akatawara Forest -41.057, 175.083; Site 2 = Tane's Track, Pakuratahi Forest -41.108, 175.154: Site 3 = Tunnel Gully, Pakuratahi Forest -41.11, 175.146: Site 4 = Reservoir Road, Wainuiomata Water Catchment -41.265, 174.991: Site 5 = Wainuiomata Recreation Area, Wainuiomata Water Catchment -41.274, 174.98: Site 6 = Cedarholm Creek, Akatawara Forest -41.018, 175.09: Site 7 = Dobson's Track, Tararua Forest Park -41.045, 175.283: Site 8 = Tunnel Gully, Pakuratahi Forest -41.104, 175.161: Site 9 = Wairongomai Track, Rimutaka Forest Park -41.252, 175.125: Site 10 = Wainuiomata Ridgeline, Rimutaka Ranges -41.269, 175.052. This image contains data sourced from the LINZ Data Service licensed for reuse under CC BY 4.0. Sourced from https://www.linz.govt.nz/land/maps/linz-topographic-maps/map-chooser/map-chooser---bq31 and https://www.linz.govt.nz/land/maps/linz-topographic-maps/map-chooser/map-chooser---bq32
